# Integrating Kolb’s experiential learning theory into nursing education: a four-stage intervention with case analysis, mind maps, reflective journals, and peer simulations for advanced health assessment

**DOI:** 10.3389/fmed.2025.1616392

**Published:** 2025-08-21

**Authors:** Jing Cheng, Yingting Wu, Li Huang, Yuehong Wu, Yuxiang Guan

**Affiliations:** ^1^School of Nursing, Anhui University of Chinese Medicine, Hefei, China; ^2^Laboratory of Geriatric Nursing and Health, Anhui University of Chinese Medicine, Hefei, China

**Keywords:** experiential learning, mind mapping, reflective practice, nursing education, competency-based education

## Abstract

**Purpose:**

This study evaluates the effectiveness of integrating case-based mind maps and reflective journals within Kolb’s experiential learning framework in advanced nursing education.

**Methods:**

An *ex post facto* design compared 2023 (control group, *n* = 46) and 2024 (experimental group, *n* = 57) cohorts of nursing master’s students. The experimental group received a Kolb-based intervention comprising: case analysis (concrete experience), reflective journals (reflective observation), mind maps (abstract conceptualization), and peer-led simulations (active experimentation). Outcomes were assessed using the Self-Directed Learning Instrument for Nursing Students (SDLINS), Critical Thinking Disposition Inventory (CTDI-CV), and a teaching satisfaction questionnaire.

**Results:**

The experimental group demonstrated significantly higher final assessment scores (96.54 ± 1.43 vs. 93.07 ± 1.62, *p* < 0.001), superior self-directed learning abilities (69.09 ± 7.56 vs. 55.63 ± 7.68, *p* < 0.001), and enhanced critical thinking skills (288.05 ± 12.41 vs. 260.13 ± 12.02, *p* < 0.001). All subdomains of these measures showed significant improvements (*p* < 0.05). Teaching satisfaction was markedly higher in the experimental group across all dimensions (*p* < 0.001), with particular strengths noted in teaching attitude (18.09 ± 2.68 vs. 11.35 ± 2.09) and classroom teaching (23.02 ± 3.59 vs. 17.83 ± 2.70).

**Conclusion:**

The systematic implementation of Kolb’s experiential learning cycle through case-based mind mapping and reflective journaling can facilitate the development of master’s nursing students’ competency in clinical advanced health assessment. This pedagogical approach effectively connects theoretical knowledge and clinical practice while fostering students’ critical thinking and self-directed learning skills.

## 1 Introduction

Nursing master’s education represents a critical stage in nurses’ careers, providing them with advanced theoretical knowledge, research skills, clinical expertise, leadership capabilities, and educational competencies. A key component of this curriculum is the “Advanced Health Assessment” course, which builds on foundational undergraduate training. This course aims to develop advanced skills in comprehensive patient evaluation, evidence-based clinical judgment, and the implementation of tailored interventions in complex clinical settings (1, 2). However, traditional lecture-based teaching methods have often failed to fully engage students, limiting their ability to meet the course’s rigorous demands. This pedagogical gap calls for the adoption of innovative strategies that align with contemporary learning theories.

Kolb’s Experiential Learning Theory (ELT) offers a robust framework for addressing these challenges. ELT posits that learning is a cyclical process comprising four stages: *concrete experience*, *reflective observation*, *abstract conceptualization*, and *active experimentation* (3). This theory integrates well with pedagogical tools such as case-based learning, mind mapping, and reflective journaling, which collectively enhance engagement and competency development in clinical education.

Case analysis, whether based on real or simulated clinical scenarios, serves as the *concrete experience* in Kolb’s cycle, immersing students in authentic clinical contexts (4). It has been shown to improve problem-solving abilities and overall competence in nursing students (5). A study by Zhang et al. showed that student-centered case studies could improve students’ critical thinking and clinical reasoning skills (4), while Waliany highlights its role in reducing cognitive biases (6). Liu et al. also demonstrated that case analysis strengthens nursing skills, competence, and student engagement in the field (5).

Mind mapping, a graphical tool for organizing knowledge, facilitates abstract conceptualization by helping students structure complex information, identify relationships, and synthesize theoretical concepts (7, 8). This aligns with Kolb’s emphasis on transforming experiences into actionable knowledge.

Reflective journals directly corresponds to the reflective observation stage, enabling students to critically analyze their learning experiences, emotions, and professional growth (9–13). This practice not only deepens understanding but also prepares students for *active experimentation*—applying refined strategies in subsequent clinical scenarios.

**Figure 1:**
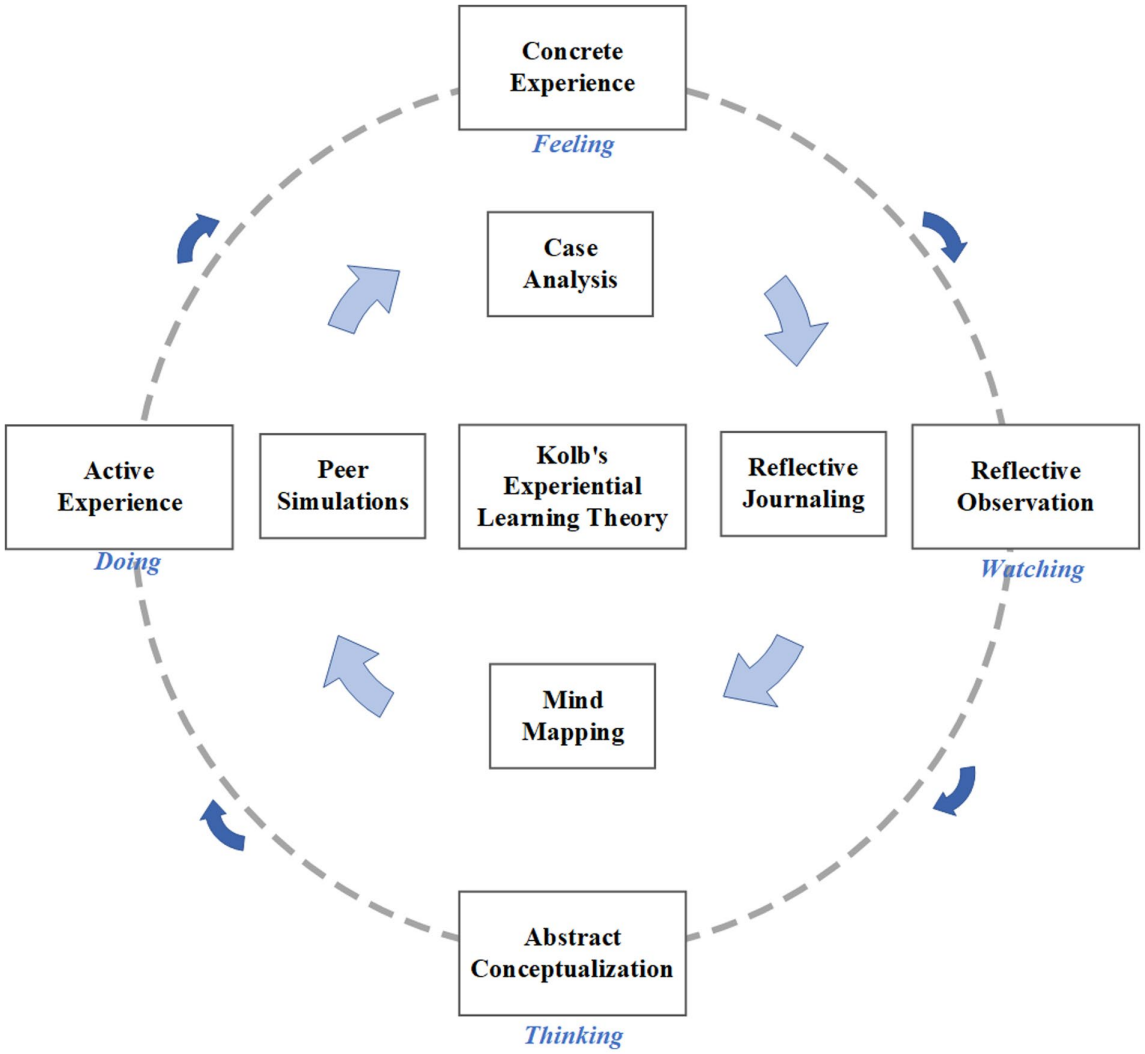
Integration of Kolb’s experiential learning cycle with four pedagogical tools. The schematic shows: Case Analysis → Concrete Experience; Reflective Journaling → Reflective Observation; Mind Mapping → Abstract Conceptualization; Peer Simulation → Active Experimentation. Arrows indicate the cyclical learning process where Active Experimentation feeds back into new Concrete Experiences.

While the effectiveness of mind mapping and reflective journaling in the education of nursing disciplines has been demonstrated (14–18), there is still less research on their relevant application in the education of the *Advanced Health Assessment* course in conjunction with their integration within the Kolb framework. This study aims to bridge this gap by investigating how the integration of case-based mind mapping and reflective journaling—guided by ELT principles—can optimize learning outcomes for nursing postgraduates (as depicted in Figure 1).

## 2 Methods

### 2.1 Ethical approval and participants

This study was approved by the Ethics Committee of Anhui University of Chinese Medicine (No. AHUCM-HSS-2024014). All participants provided written informed consent. Consent forms detailed study objectives, voluntary participation, and data anonymity, following Ethics Committee guidelines. Participants included two cohorts of nursing master’s students: the 2023 cohort (*n* = 46) as the control group (traditional teaching) and the 2024 cohort (*n* = 57) as the experimental group (Kolb-based intervention). Both groups were taught by the same faculty, used identical materials, and followed matched schedules to ensure baseline comparability between groups.

### 2.2 Intervention design based on Kolb’s experiential learning cycle

#### 2.2.1 Control group

The control group received teacher-centered instruction comprising lectures using standardized slides aligned with core course content, complemented by weekly small-group discussions (4–5 students per group) focused on textbook-based case analyses to practice clinical assessment strategies. Additionally, students completed biweekly written assignments (e.g., clinical guideline critiques, symptom differentiation tasks) and a final examination assessing competencies through multiple-choice questions (60%), clinical reasoning scenarios (30%), and Objective Structured Clinical Examination (OSCE)-style skills evaluations (10%), including history-taking, physical examination, and diagnostic prioritization. This approach balanced theoretical knowledge delivery with practical application while maintaining alignment with course objectives.

#### 2.2.2 Experimental group

The experimental group’s intervention was rigorously designed around Kolb’s experiential learning cycle, with faculty receiving specialized training to ensure fidelity. This training covered both theoretical foundations and practical implementation, including: (1) aligning activities with each learning stage (e.g., case selection for concrete experience), and (2) using standardized rubrics to assess mind maps (abstract conceptualization) and reflective journals (reflective observation).

##### 2.2.2.1 Case development

Faculty-researcher teams developed authentic clinical cases [e.g., chronic obstructive pulmonary disease (COPD), acute myocardial infarction] adapted from real patient records, explicitly mapped to Kolb’s stages. Case complexity progressed with student skill development, integrating theoretical knowledge (e.g., pathophysiology) with practical application to ensure seamless transitions between learning phases.

##### 2.2.2.2 Classroom implementation

The intervention followed a structured weekly cycle:

1. Pre-class preparation

One week before each session, student teams (5–6 members) received cases via *Rain Classroom*, collaborating to create preliminary hierarchical mind maps (*MindManager®*) identifying clinical data relationships.

2. In-class activities

- Concrete experience: 30-min case study tutorial (e.g., COPD exacerbation diagnosis).
- Reflective observation: 20-min structured journals to record diagnostic uncertainties/insights.
- Abstract conceptualization: 40-min mind map refinement into care plans with real-time faculty feedback.
- Active experimentation: 30-min peer-led care plan debates and simulations.

3. Post-class consolidation

Journal sharing sessions consolidated learning through peer reflection.

**Figure 2:**
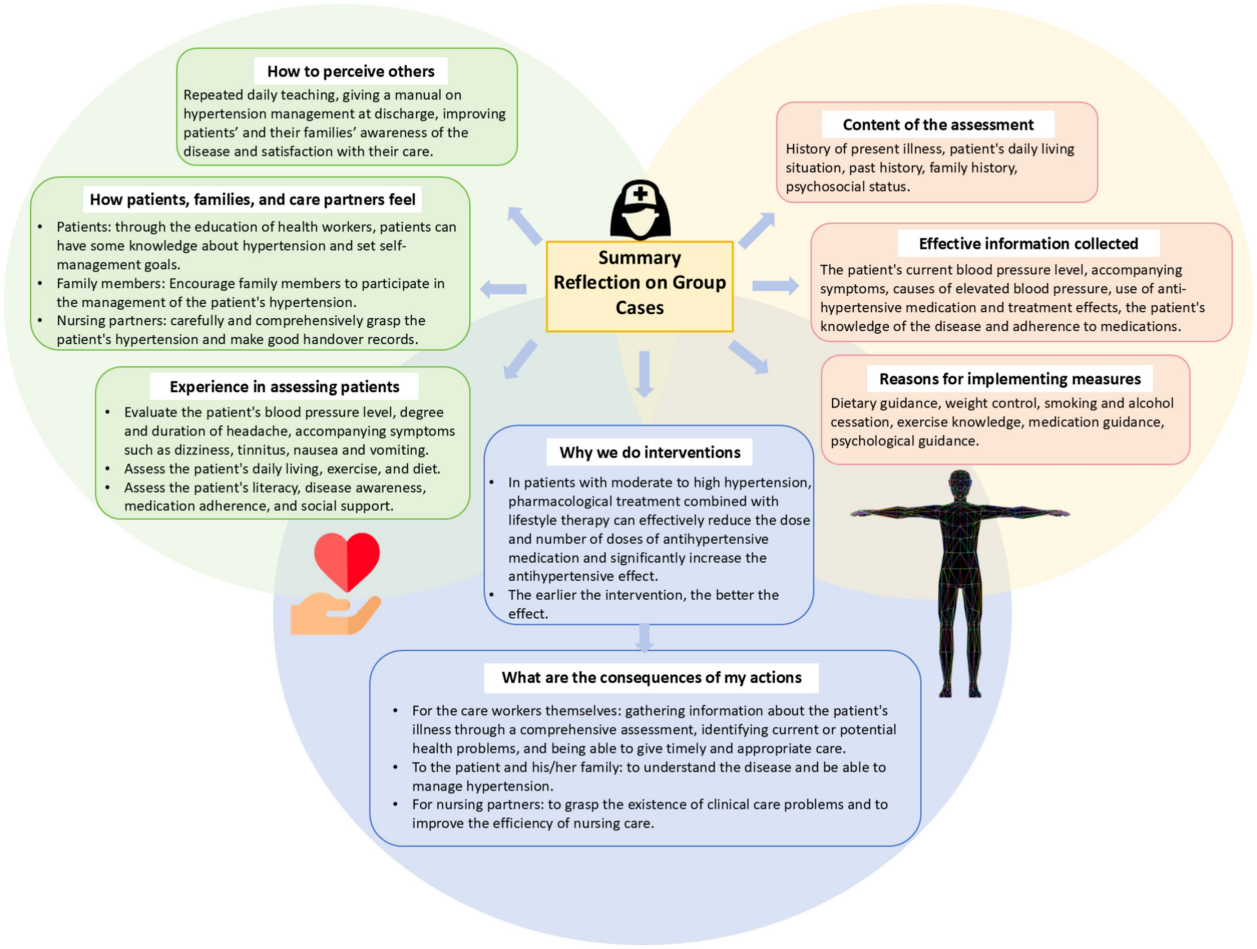
An example for case-based mind map and reflective journal in advanced health assessment. The sample mind map (left panel) illustrates how students synthesized hypertension management knowledge into a hierarchical structure, supporting *abstract conceptualization* by visually linking pathophysiology to clinical interventions. The reflective journal (right panel) prompted learners to document diagnostic uncertainties (e.g., “How did the patient’s medication adherence influence outcomes?”), fostering *reflective observation* through critical self-evaluation. Together, these tools operationalized Kolb’s cyclical learning process, enabling iterative transitions between experience and application.

A three-pronged assessment strategy aligned with Kolb’s phases (Figure 2):

- Mind maps assessed conceptual integration (abstract conceptualization).
- Reflective journals evaluated critical reflection (reflective observation).
- Group presentations measured practical application (active experimentation).

### 2.3 Evaluation of teaching effectiveness

The evaluation framework explicitly aligned assessment components with Kolb’s experiential learning stages.

#### 2.3.1 Course scores

Final exam performance was compared between the cohorts. The assessment rubric focused on competencies in active experimentation (e.g., care plan formulation, clinical prioritization) and abstract conceptualization (e.g., integration of pathophysiology), in line with Kolb’s emphasis on applying knowledge.

#### 2.3.2 Self-directed learning ability

The Self-Directed Learning Instrument for Nursing Students (SDLINS) assessed competencies related to Kolb’s reflective observation and active experimentation stages (19). The SDLINS consists of 20 items across four dimensions: learning motivation, planning and implementation, self-monitoring, and interpersonal communication. Higher scores indicated stronger self-directed learning abilities, with a Cronbach’s *α* of 0.916.

#### 2.3.3 Critical thinking ability

The Chinese version of the Critical Thinking Disposition Inventory (CTDI-CV) assessed critical thinking abilities, central to Kolb’s abstract conceptualization and reflective observation stages (20). The scale includes seven dimensions: seeking truth, open-mindedness, analytical ability, systematic thinking, confidence, curiosity, and cognitive maturity. Higher scores indicated better integration of experiential learning into critical reasoning, with a content validity of 0.89 and Cronbach’s *α* of 0.90.

#### 2.3.4 Teaching satisfaction questionnaire

A 22-item questionnaire evaluated student satisfaction with teaching. The questionnaire covered five domains: teaching preparation, classroom teaching, teaching ability, teaching attitude, and teaching effectiveness. Items were rated on a 5-point Likert scale, with higher scores indicating greater satisfaction. The content validity index was 0.91, and a pilot study demonstrated excellent reliability (Cronbach’s *α* = 0.927).

### 2.4 Statistical analysis

The quantitative data were expressed as mean ± standard deviation ( $\bar{\chi }$ ± s). The comparison between the two groups was conducted using an independent samples *t*-test. All statistical analyses were performed using SPSS 13.0 software, and a *p* value less than 0.05 was considered statistically significant.

## 3 Results

### 3.1 Course performance

Baseline demographic characteristics, including age (24.26 ± 2.53 vs. 24.82 ± 2.58, *p* = 0.135), gender distribution (7 vs. 4 males, *p* = 0.208), and entrance examination scores (74.48 ± 3.10 vs. 75.59 ± 4.34, *p* = 0.150), showed no significant differences between the 2023 (control) and 2024 (experimental) cohorts, confirming group comparable at baseline (Table 1).

**Table 1:** Comparisons of age, gender composition, and entrance exam scores between groups.

Characteristic	Control group	Experimental group	*t*/*χ*^2^	*p*
Age (years)	24.26 ± 2.53	24.82 ± 2.58	1.552	0.135
Gender [Male, n (%)]	7 Male	4 Male	1.878	0.208
Entrance exam score	74.48 ± 3.10	75.59 ± 4.34	−1.502	0.150

The experimental group, which received the Kolb-based pedagogical intervention, achieved significantly higher final assessment scores (96.54 ± 1.43) compared to the control group (93.07 ± 1.62) (*t* = 8.731, *p* < 0.001). This improvement underscores the efficacy of experiential learning in advancing clinical competency among nursing students.

### 3.2 Self-directed learning ability aligned with Kolb’s experiential learning stages

The experimental group demonstrated significant improvements in self-directed learning (SDL) across all stages of Kolb’s experiential learning cycle, as detailed in Table 2.

**Table 2:** Post-course self-directed learning ability aligned with Kolb’s experiential learning stages.

Kolb’s stage	Dimension	Control group	Experimental group	*t*	*p*	95% CI	*η* ^2^
Concrete Experience	Learning motivation	15.33 ± 3.03	17.58 ± 2.99	−3.783	<0.001	−3.07, −3.32	0.217
Reflective observation	Self-monitoring	11.96 ± 3.39	15.26 ± 3.68	−4.699	<0.001	−4.53, −1.40	0.285
Abstract conceptualization	Planning and implementation	16.17 ± 3.40	19.28 ± 5.53	−3.333	0.001	−4.19, −0.56	0.271
Active experimentation	Interpersonal communication	12.17 ± 5.13	16.96 ± 3.04	−7.065	<0.001	−6.98, −3.17	0.403
Total		55.63 ± 7.68	69.09 ± 7.56	−8.913	<0.001	−15.26, −8.97	0.639

#### 3.2.1 Concrete experience (learning motivation)

Students in the experimental group exhibited markedly higher learning motivation compared to the control group (17.58 ± 2.99 vs. 15.33 ± 3.03, *p* < 0.001; Table 2).

#### 3.2.2 Reflective observation (self-monitoring)

Structured reflective journals significantly enhanced self-monitoring abilities in the experimental group (15.26 ± 3.68 vs. 11.96 ± 3.39, *p* < 0.001; Table 2). Students critically evaluated their clinical reasoning processes, identifying gaps and adapting strategies iteratively.

#### 3.2.3 Abstract conceptualization (planning and implementation)

The experimental group outperformed the control group in planning and implementation (19.28 ± 5.53 vs. 16.17 ± 3.40, *p* = 0.001; Table 2), reflecting their ability to transform structured knowledge from mind mapping exercises into actionable care plans.

#### 3.2.4 Active experimentation (interpersonal communication)

Collaborative tasks, such as peer-led simulations and debates, led to superior interpersonal communication skills in the experimental group (16.96 ± 3.04 vs. 12.17 ± 5.13, *p* < 0.001; Table 2). This underscores the role of active experimentation in bridging theory and practice.

These findings underscore Kolb’s framework as a robust tool for cultivating SDL, where experiential engagement and reflective refinement synergistically enhance nursing competencies.

### 3.3 Critical thinking development through Kolb’s experiential learning cycle

The experimental group exhibited significant improvements in critical thinking across all stages of Kolb’s experiential learning cycle, as demonstrated in Table 3.

**Table 3:** Post-course critical thinking ability aligned with Kolb’s experiential learning stages.

Kolb’s stage	Dimension	Control group	Experimental group	*t*	*p*	95% CI	*η* ^2^
Concrete experience	Seeking truth	35.30 ± 6.09	38.74 ± 6.74	−2.682	0.009	−5.90, −0.44	0.252
Reflective observation	Open mindedness	35.89 ± 5.42	40.21 ± 6.03	−3.781	<0.001	−5.34, −0.73	0.410
Abstract conceptualization	Analytical ability	40.46 ± 4.83	43.39 ± 4.33	−3.241	0.002	−4.81, −0.57	0.231
	Systematic thinking	35.59 ± 5.46	38.88 ± 3.76	−3.615	0.001	−4.73, −0.75	0.261
Active experimentation	Confidence in critical thinking	39.57 ± 4.32	43.82 ± 4.43	−4.904	<0.001	−5.21, −1.41	0.268
	Curiosity	40.48 ± 5.60	43.25 ± 5.65	−2.481	0.015	−5.88, −1.07	0.233
Reflective ↔ Abstract	Cognitive maturity	32.85 ± 4.36	39.77 ± 4.39	−7.984	<0.001	−7.86, −4.14	0.519
Total		260.13 ± 12.02	288.05 ± 12.41	−11.513	<0.001	−29.67, −19.67	0.732

#### 3.3.1 Concrete experience (seeking truth)

Engagement with authentic clinical cases (e.g., COPD, acute myocardial infarction) enhanced evidence-seeking behaviors in the experimental group, with scores for *seeking truth* surpassing the control group (38.74 ± 6.74 vs. 35.30 ± 6.09, *p* = 0.009).

#### 3.3.2 Reflective observation (open-mindedness)

Structured reflective journals fostered integrative analysis of conflicting clinical data, leading to superior *open-mindedness* in the experimental group (40.21 ± 6.03 vs. 35.89 ± 5.42, *p* < 0.001).

#### 3.3.3 Abstract conceptualization (analytical and systematic thinking)

Mind mapping exercises significantly strengthened both *analytical ability* (43.39 ± 4.33 vs. 40.46 ± 4.83, *p* = 0.002) and *systematic thinking* (38.88 ± 3.76 vs. 35.59 ± 5.46, *p* = 0.001), validating the role of abstract conceptualization in organizing complex clinical knowledge.

#### 3.3.4 Active experimentation (confidence and curiosity)

Peer-led simulations and hypothesis-testing debates elevated *confidence in critical thinking* (43.82 ± 4.43 vs. 39.57 ± 4.32, *p* < 0.001) and sustained *curiosity* (43.25 ± 5.65 vs. 40.48 ± 5.60, *p* = 0.015), highlighting the impact of iterative clinical application.

#### 3.3.5 Dynamic interaction (cognitive maturity)

The experimental group showed significantly higher cognitive maturity scores compared to the control group (39.77 ± 4.39 vs. 32.85 ± 4.36; *p* < 0.001, Table 3).

These robust findings demonstrate that Kolb’s cycle functions both as an effective pedagogical approach and as a cognitive scaffold for clinical expertise development. The systematic linkages of concrete experiences with structured reflection and practical application prepares nursing students to effectively manage healthcare complexities.

### 3.4 Teaching satisfaction aligned with Kolb’s experiential learning cycle

The experimental group reported significantly higher satisfaction across all dimensions of the Kolb-based pedagogical intervention, as detailed in Table 4. Below, we contextualize these findings within each stage of the experiential learning cycle:

**Table 4:** Post-course critical thinking ability aligned with Kolb’s experiential learning stages.

Kolb’s stage	Dimensions	Control group	Experimental group	*t*	*p*	*η* ^2^
Concrete experience	Classroom teaching (Case Immersion)	17.83 ± 2.70	23.02 ± 3.59	−8.364	<0.001	0.491
Reflective observation	Teaching ability (Guided Reflection)	11.63 ± 2.95	18.40 ± 2.99	−11.059	<0.001	0.738
Abstract conceptualization	Teaching preparation	11.24 ± 1.95	13.89 ± 1.93	−6.910	<0.001	0.384
Active experimentation	Classroom teaching (Peer Simulation)	17.83 ± 2.70	23.02 ± 3.59	−8.364	<0.001	0.491
Full cycle integration	Teaching effectiveness	13.00 ± 1.93	16.23 ± 2.82	−6.868	<0.001	0.350
Cyclical commitment	Teaching attitude	11.35 ± 2.09	18.09 ± 2.68	−13.964	<0.001	0.764

#### 3.4.1 Abstract conceptualization (teaching preparation)

Faculty efforts to align instructional materials with theoretical frameworks (e.g., mind mapping for COPD pathophysiology) were rated markedly higher by the experimental group (13.89 ± 1.93 vs. 11.24 ± 1.95, *p* < 0.001). Students emphasized how structured knowledge organization facilitated seamless transitions between theory and practice.

#### 3.4.2 Concrete experience (case immersion)

Immersive case discussions simulating real-world clinical environments (e.g., sepsis protocols in ICU rounds) received the highest satisfaction ratings (23.02 ± 3.59 vs. 17.83 ± 2.70, *p* < 0.001), validating the role of authentic experiential engagement.

#### 3.4.3 Reflective observation (guided reflection)

Faculty expertise in facilitating reflective journals and providing actionable feedback drove exceptional satisfaction with teaching ability (18.40 ± 2.99 vs. 11.63 ± 2.95, *p* < 0.001). Students highlighted the value of iterative self-evaluation in refining clinical reasoning.

#### 3.4.4 Active experimentation (peer simulation)

Collaborative peer-led debates and low-risk hypothesis testing significantly enhanced satisfaction with active experimentation (23.02 ± 3.59 vs. 17.83 ± 2.70, *p* < 0.001), underscoring the importance of safe environments for clinical application.

#### 3.4.5 Full cycle integration (teaching effectiveness)

Students recognized the intervention’s success in operationalizing Kolb’s complete cycle, with teaching effectiveness ratings significantly higher in the experimental group (16.23 ± 2.82 vs. 13.00 ± 1.93, *p* < 0.001).

#### 3.4.6 Cyclical commitment (teaching attitude)

Faculty responsiveness to student input—evidenced by weekly case refinements based on reflections—yielded the highest satisfaction (18.09 ± 2.68 vs. 11.35 ± 2.09, *p* < 0.001), reflecting a shared commitment to iterative learning.

These robust findings demonstrate that the observed satisfaction reflects not merely enjoyment, but rather a fundamental alignment between the pedagogical approach and Kolb’s experiential learning theory, validating the intervention’s educational efficacy.

## 4 Discussion

### 4.1 Case-based mind maps and reflective journals enhance learning effectiveness through Kolb’s experiential learning cycle

The experimental group’s superior academic performance aligns with Kolb’s ELT, where competency development emerges through iterative engagement with four stages: *concrete experience*, *reflective observation*, *abstract conceptualization*, and *active experimentation* (21). Our intervention uniquely operationalized this cycle by integrating case analyses, reflective journals, and mind mapping into a synergistic pedagogical ecosystem. Immersive case analyses (e.g., COPD exacerbation, acute MI management) provided authentic diagnostic challenges, fostering problem-solving skills consistent with contextualized learning principles (4). However, unlike prior studies using isolated tools, we explicitly structured transitions between Kolb’s stages—students progressed from case immersion to reflection and conceptualization, highlighting the insufficiency of case analysis alone without cyclical scaffolding (22). Reflective journals, guided by standardized prompts enabled iterative refinement of clinical reasoning, contrasting with the limited efficacy of unstructured journaling in nursing education (10). Mind mapping further transformed fragmented knowledge into hierarchical frameworks (e.g., COPD, pathophysiology to interventions), aligning with visual tools’ role in conceptual integration (8), while innovating through peer feedback-driven revisions. Finally, peer-led simulations and debates created low-risk hypothesis-testing environments, mirroring OSCE benefits (23) but emphasizing collaborative social learning. Crucially, the cyclical interplay of these tools—where reflection informed conceptualization (e.g., journals refining mind maps) and experimentation generated new experiences (e.g., peer feedback shaping subsequent cases)—formed a self-reinforcing loop absent in traditional instruction, explaining the experimental group’s marked academic gains.

### 4.2 Self-directed learning (SDL) as a manifestation of cyclical engagement

The experimental group demonstrated significant improvements across all SDL dimensions, robustly supporting Kolb’s proposition that cyclical engagement in experiential learning fosters autonomous learning capabilities. Notably, learning motivation was enhanced through the synergistic interplay of case-based immersion (concrete experience) and peer feedback (active experimentation), contextualizing theoretical knowledge in clinical practice—a finding aligned with SDL principles emphasizing learner agency (24, 25). This aligns with the immersive case analyses and peer feedback activities, which contextualized theoretical knowledge within authentic clinical scenarios. The substantial improvement in planning and implementation emerged from mind mapping’s role in abstract conceptualization, enabling students to transform knowledge into actionable workflows through iterative revisions based on peer input—an adaptation absents in earlier visual scaffolding research (26). Similarly, reflective journaling facilitated self-monitoring by guiding students to evaluate clinical reasoning gaps, addressing critiques of unstructured journaling’s limited efficacy (27). These findings collectively validate Knowles’ adult learning principles (28), demonstrating that SDL flourishes when educational approaches emphasize active experiential engagement rather than passive knowledge transmission. Particularly noteworthy is the exceptional progress in interpersonal communication skills, underscoring how collaborative active experimentation fosters essential teamwork competencies alongside cognitive development (29).

### 4.3 Critical thinking development through experiential scaffolding

Critical thinking refers to the ability to analyze, evaluate, and interpret information logically and systematically (30). The experimental group’s superior performance in critical thinking demonstrates the efficacy of Kolb’s experiential framework in cultivating higher-order cognitive skills essential for clinical practice. These gains, observed across all CTDI-CV dimensions, underscore how structured engagement with clinical complexity through Kolb’s cycle fosters robust critical thinking. Enhanced analytical ability and systematic thinking emerged from mind mapping’s role in abstract conceptualization, enabling students to deconstruct cases into logically organized frameworks—a finding aligned with Lin et al.’s emphasis on visual scaffolding but extending it through iterative peer-driven revisions (31). Similarly, reflective journaling promoted open-mindedness and cognitive maturity by challenging assumptions and integrating diverse perspectives, resonating with interdisciplinary studies on structured reflection (32). Active experimentation further elevated confidence and curiosity through peer debates and simulated OSCEs, highlighting how low-risk hypothesis testing nurtures both willingness and capacity for critical analysis (21). Crucially, these outcomes illustrate that critical thinking arises not from isolated tools but from the integrated cyclicality of Kolb’s stages: concrete experiences provided diagnostic challenges, reflection refined reasoning, conceptualization structured knowledge, and experimentation tested hypotheses. The cyclical interplay between reflective observation and abstract conceptualization drove substantial gains in *cognitive maturity*, demonstrating the non-linear nature of clinical reasoning development. This dynamic interplay created a reinforcing cognitive ecosystem where analytical precision, intellectual flexibility, and evidence-seeking behaviors mutually enhanced clinical decision-making—a transformative outcome unattainable through traditional didactic methods (33).

### 4.4 Case-based mind maps and reflective journals enhance teaching satisfaction

The experimental group’s consistently high satisfaction ratings across all dimensions demonstrate the profound impact of Kolb’s experiential approach on the learning experience. Students reported particularly strong satisfaction with classroom instruction, where the dynamic integration of case discussions, reflective journaling, mind mapping, and peer simulations created an engaging, clinically relevant environment. Quantitative ratings underscored the explicit connection between theory and practice, mirroring established findings on contextualized learning’s role in nursing education (34). Exceptional satisfaction with teaching ability highlighted a collaborative culture where weekly case refinements based on student input fostered perceptions of partnership. High scores for teaching preparation and overall effectiveness further confirmed that systematically implementing Kolb’s cycle across instructional components addressed diverse learning needs without compromising rigor. These results transcend conventional satisfaction metrics, revealing how faithful operationalization of experiential principles—through sequenced concrete experiences, reflection, conceptualization, and application—created a self-reinforcing educational ecosystem (35). The pedagogy not only elevated perceived teaching quality but also deepened engagement and competency development, validating its transformative potential in advanced nursing education.

### 4.5 Limitations

While this study demonstrates the efficacy of Kolb’s ELT in clinical education, several limitations must be acknowledged. First, the single-institution setting may limit generalizability, as institution-specific factors (e.g., faculty expertise, curricular structure) could influence outcomes. Second, potential cohort effects—such as unmeasured yearly variations in clinical exposure or student motivation—may confound results despite baseline comparability. Third, the absence of long-term follow-up precludes conclusions about the durability of competency retention post-graduation.

Methodological and theoretical constraints further warrant consideration. Kolb’s linear cyclical structure assumes sequential progression through stages, potentially oversimplifying clinical learning contexts requiring non-linear, adaptive problem-solving (36). Additionally, individual differences in learning styles (e.g., stage-specific preferences) may lead to uneven engagement, challenging ELT’s universal applicability (37). Finally, the resource-intensive nature of Kolb-based interventions—requiring faculty training, structured case development, and sustained reflective activities—poses scalability challenges in resource-constrained settings (38).

## 5 Conclusion

This study demonstrates that systematically integrating case-based mind maps and reflective journals within Kolb’s experiential learning framework significantly enhances clinical competency development in advanced nursing education. The intervention’s success is evidenced by the experimental group’s superior academic performance, significant higher scores in self-directed learning and critical thinking, and consistently high teaching satisfaction—outcomes attributable to the cyclical interplay of Kolb’s stages: clinical immersion (concrete experience), guided reflection (reflective observation), mind mapping (abstract conceptualization), and peer-led simulations (active experimentation). This approach creates a dynamic learning ecosystem by linking theory and practice through engaging iteratively. In this ecosystem, precise analysis, adaptive reasoning, collaborative problem solving, and mutual reinforcement can strengthen the clinical expertise of master’s degree nursing students.

## Data Availability

The raw data supporting the conclusions of this article will be made available by the authors, without undue reservation.
